# The Social Course of Fibromyalgia: Resisting Processes of Marginalisation

**DOI:** 10.3390/ijerph19010333

**Published:** 2021-12-29

**Authors:** Nicole Brown

**Affiliations:** UCL Institute of Education, College London, London WC1H 0AL, UK; nicole.brown@ucl.ac.uk

**Keywords:** illness experience, fibromyalgia, UK, resistance, marginalisation, fibromyalgia syndrome, interviews, metaphorical understanding, embodiment

## Abstract

This sociological article reports an empirical study into the lived experience of fibromyalgia. It includes 28 participants (26 women, 2 men) with a formal diagnosis of fibromyalgia. Data collection consisted of the completion of an identity box project and subsequent interviews. Data analysis followed the principles of iterative, inductive, semantic thematic analysis, and led to the identification of four major themes: the role of the social in making sense of the experience, the process of redefining lifegoals, the refusal to accept fibromyalgia as a diagnosis, and the consideration of identifying as a patient. These themes in turn demonstrate four forms of resistance against processes of marginalisation amongst those who have been diagnosed with fibromyalgia: (1) the incorporation of societal expectations and norms into their life-stories; (2) the re-making the lifeworld at a cerebral level through redefining reality and creating a new, socially acceptable reality; (3) the active rejection of the fibromyalgia diagnosis; and (4) the employment of active and pro-active countermeasures to assuming the sick role.

## 1. Introduction

Fibromyalgia is not a disease or an illness. Fibromyalgia is characterised by chronic, wide-spread pain, fatigue, sleep disturbances, cognitive dysfunctions, often described as “brain fog” or “fibro fog”, increased sensitivity and psychological disorders [1] and it is associated with a wide range of somatic symptoms [2]. Fibromyalgia is a syndrome, a collection of symptoms that is often only diagnosed by excluding other conditions in combination with consideration of pain experiences and the presence of tender points. The lack of a definite process for diagnosis and the variability of the condition make it a doubtful and contested condition [3,4,5,6] with nowadays largely two opposing camps amongst the medical professionals: those who believe that there is an organic and physiological cause to the condition and who therefore continue to search for underlying triggers or biomarkers [2,7] and those who see fibromyalgia as a psychogenic, psychosomatic, or behavioural condition [8,9,10]. Consequently, pain scales and symptomatic variation amongst individuals with fibromyalgia are also considered in different ways depending on the researchers’ positionality vis-á-vis the syndrome.

As fibromyalgia research has grown drastically over the past two decades, the lived experience of fibromyalgia is well-documented with researchers focussing on the experience of receiving and coming to terms with the fibromyalgia diagnosis [11,12,13], the overall illness experience of fibromyalgia [14,15,16,17,18] or more specifically, the experience of stigmatisation [19,20], the relationship of fibromyalgia patients and their significant others or health care professionals [21,22], the role of support networks and systems in the experience of fibromyalgia [23,24,25], as well as identity and loss of self [21,26]. Clearly, there is now a recognition that in line with a sociosomatic understanding of illness, experiences of physical pain in fibromyalgia are a manifestation of emotional pain, stresses and traumas [27]. However, this is only part of the story. Fibromyalgia prevalence studies confirm a male to female ratio of 1:9 [28], with the large majority of women diagnosed with fibromyalgia aged from 30 to 40 years and older. There is ample evidence for women to engage in more health-seeking behaviours and to pursue diagnosis more fervently than men, who are known to withdraw into addiction, alcoholism, and develop anger management issues instead of seeking help with doctors [29]. In addition, some medical research suggests that there may be a link between fibromyalgia and fibromyalgia-like symptoms and hormone levels in the human body, which would indeed result in more women being diagnosed with the condition [30]. Nonetheless, the role of hormones in fibromyalgia and in diseases in general remains under-explored. As critics highlight, medical research considers sex/gender as an “irritant variable” [31], and so continually fails to account for the specificities of the female body. Yet, differences in gender cannot solely be explained biologically [32] and therefore require a more social constructionist view that would consider individuals’ social environment [33] and the impact of individuals’ social environment on gender and health behaviours as well as coping mechanisms [34]. The genderisation of the condition and the entire medical field combined with the lack of specific research considering the female body by comparison to the male body is one of the major criticisms of contemporary fibromyalgia research. On the one hand, (bio)medical perspectives emphasise that women show more increased somatic tendencies than men; on the other hand, the entire process to identify symptomology and develop classifications for the fibromyalgia diagnosis started with the determination of tender points, which are more pronounced in women due to their heightened somatic tendencies. In this sense, the fibromyalgia diagnosis represents an attempt to fit women’s distress and somaticism into a neat (bio)medical order instead of a distinct, uncontested and uncontextualised diagnostic unit [31]. So far, the role of the social in the illness experience of fibromyalgia has remained underexplored.

The role of society and cultural environment within the context of and understanding of illness and illness narratives is well documented [35,36,37] in medical anthropological and sociological discourses, and increasingly within the medical realm [38]. Within these conceptualisations, illness is seen as constructed at three different levels, as it is embedded with cultural meaning, but also defined at an experiential level and shaped by the medical discourses [39]. Illness symptoms which individuals experience are felt as sensations, but also experienced at an emotional, embodied level as learnt responses to conventions [40]. In this sense, illness symptoms are physical manifestations of societal ills and cultural influences [35] or a lived experience placed within a society or culture [37]. In Ware’s [37] terms the qualities of distress experienced at an individual level combined with cultural expectations, societal norms and conventions result in processes of marginalisation: individuals are faced with delegitimisation of their illness experiences, with limitations to their activities and thus to their role performance, with financial consequences and social isolation. In order to prevent this marginalisation and being pushed to the edges of their social group, resistance strategies are employed.

Amongst people diagnosed with chronic fatigue syndrome, Ware [37] describes preserving and re-making the lifeworld as two groups of resistance strategies that are commonly performed as maintaining existing activities, even at the expense of experiencing symptom aggravation, or pacing themselves through cutting down on what are considered as less important activities [37]. Further resistance strategies include educating others [41,42], distancing oneself from the illness or condition [43], trivialising the condition [44], strategic avoidance of particular situations and conversations, or defiance in view of the inevitable [45], which are all simplified in a model describing resistance behaviours as strategies to deflect versus challenge or confront [46]. In this article, I draw on sociological theory and use Ware’s model to explore the social course of fibromyalgia amongst academic women diagnosed with fibromyalgia. In doing so, this article pursues two aims: firstly, to highlight the difficulties of navigating an invisible and fluctuating chronic condition in an environment that emphasises productivity and excellence; and secondly, to shed light on strategies and techniques individuals employ to negotiate their lives with fibromyalgia and counteract marginalisation.

## 2. Methodology and Methods

### 2.1. Context and Research Question

The overarching aim of the research was to explore the relationship between fibromyalgia as a contested condition and the construction of academic identity, in particular in view of the cognitive dysfunctions linked to fibromyalgia that would potentially be at odds with the cerebral and scholarly work academics are expected to undertake; to offer insights into academia as a workplace; to identify how this particular workplace may foster or potentially cause distress that becomes physically manifested in a disabling condition; and to investigate how individuals counter processes of marginalisation within this particular working environment. To this end, the following research question and subsidiary questions were formulated:

What is the relationship between fibromyalgia and academic identity?
What is the role of academia for an academic?
What does fibromyalgia mean for an academic?
How is fibromyalgia experienced in academia?

### 2.2. Recruitment and Sampling

Once full ethical approval was gained from the University of Kent, UK, ethics committee, participants who identified as academics with fibromyalgia were recruited via three different recruitment strategies: via fibromyalgia support groups in the South East of England, through direct mailing via the equality, disability, inclusion and wellbeing teams at universities across the UK and via presentations of research posters at national conferences. A QR code on the poster led viewers directly to a specific area on a personal web site, which would provide more information. The poster also contained direct contact details and included a pocket with flyers and business cards for those interested in the research to take away. As a result, potential participants, who self-identified as academics in the UK and who had had a fibromyalgia diagnosis asked to be part of the research.

For participants to be eligible for the research they needed to have been diagnosed with fibromyalgia by health care professionals in the UK context, and they needed to self-identify as academics. This led to the sample size being varied in terms of the academic positions, roles and workplaces represented. Academic roles ranged from early careers researchers currently undertaking their PhD studies, to mid-career academic practitioners and lecturers to professors in later career stages. Participants’ working conditions ranged from independent research positions and self-employment to hourly paid lecturers, part- and full-time employments in Further Education and Higher Education contexts. The fibromyalgia diagnosis as inclusion criterion was more clearly defined and led to fewer variabilities. All participants had received a formal diagnosis at some point in their lives, with the time lived with a formal fibromyalgia diagnosis between 3 and 10 years.

In the end, 28 participants (26 women, 2 men) were recruited. The following table (Table 1) outlines the classification of the participants according to their career stages, their employment statuses, and their institutional contexts.

During the course of the research, two participants’ fibromyalgia diagnoses were re-evaluated and refined. The fibromyalgia diagnosis was not entirely revoked but reframed within the context of Ehlers–Danlos Syndrome and Central Sensitisation Disorder, respectively. As the fibromyalgia diagnosis continued to be relevant and both participants’ treatments for fibromyalgia were also continued, the participants’ contributions to the research were not excluded. Additionally, 8 participants, indicated by an asterisk, dropped out. Their stories and input up to the point of dropping out were very relevant for this study and were used in the analysis.

### 2.3. Data Generation

This qualitative sociological research was an Embodied Inquiry [47] based on a participatory, interpretivist approach [48] that drew on principles of arts-based research in order to account for three basic principles: (1) Human language is limited and limiting, especially when individuals try to explain and describe sensations, such as pain, or other embodied and bodily experiences [49,50,51,52]. (2) Related to the limitation of language, human understanding and experiences are fundamentally embodied [53]. (3) Due to the embodiedness of human understanding and the arbitrariness of language, humans turn to metaphorical expressions and forms of communication in order to compensate [54]. As a consequence, data collection was consciously designed in such a way that the measures commonly used in medical settings, such as pain scales and symptom mapping, would not be employed. Instead, data collection consisted of the completion of an identity box project [55] and subsequent interviews. As part of the identity box project participants were asked to respond to five individual questions by choosing objects to put into a box, taking a photograph of the box with the objects and briefly commenting on the objects and their meanings. The five questions were “Who are you?”, “What affects you?”, “How do others see you?”, “What role does fibromyalgia play?”, and “What is life with fibromyalgia like?”. Once the project was completed, semi-structured interviews were scheduled to be held via Skype calls. The interviews were conceived as conversations or interactions between participants and the researcher [56] that represented a meaning-making process in which the researcher makes sense of the participants making sense of their experiences [57]. Interviews were semi-structured to allow for open and spontaneous responses [58], but were focussed on exploring the lived experience of fibromyalgia under the influence of fibromyalgia. Interviews lasted between 50 and 60 min. In line with the study’s theoretical framework, participants were encouraged to find their preferred form of communication and expression, and were asked for consent, assent, and continued participation at each stage of the research following the principles of process ethics in participatory research designs [59].

### 2.4. Analysis

All interviews were transcribed verbatim and entered into NVivo for coding according to the principles of thematic analysis [60] in its intended reflexive form of research practice [61] combined with analytical approaches commonly used within and borrowed from visual methodologies [62,63]. In this systematic visuo-textual analysis [64], visuals and textual data sets were initially reviewed in isolation of one another (see Table 2). As a first step, the visual materials were coded with descriptive, organisational, and conceptual observations [57]. Subsequently, the textual data from emails were also subjected to this first coding before the data were combined to provide a third level of analysis, one where the textual and visual data were considered in conjunction with one another.

The application of the systematic visuo-textual analysis [64] resulted in an iterative spiral process that allowed for a deepening of understanding of data on a single and multi-layered level. In effect, the analysis represented a vertical weave from element 1 level 1 to element 1 level 2 back to level 1, before element 2 levels 1 and 2 were approached in the same way. The final weave was from element 3 level 1 to level 2 and back to level 1. All steps and stages were observed in order to ensure robustness of the analysis. Table 3 and Table 4 are two examples of how data was analysed at participant-level, and how similar topics were discussed in the conversations and represented through object work.

From this iterative process of consistently weaving in and out of data sets associated with individual participants across the entirety of all visual and textual data four themes were identified: (1) the role of the social in making sense of the experience, (2) the process of redefining life goals, (3) the refusal to accept fibromyalgia as a diagnosis, and (4) the consideration of identifying as a patient and assuming the sick role. In the following section, the findings will be presented in these four themes, which were subsequently related to Ware’s [37] social course of illness to identify academics’ behaviours and strategies to counteract experiences of marginalisation through having fibromyalgia.

## 3. Findings

### 3.1. The Role of the Social in Making Sense of the Experience

At a personal and emotional level, participants feel it difficult to come to terms with a condition that is not clear-cut and commonly accepted or widely understood. Participants struggle to make complete sense of the label and their experiences. What academics in this study said about their experiences with fibromyalgia and being diagnosed demonstrates the complexity of coming to terms with a condition more generally on a personal level. The overarching sentiment is one of confusion, uncertainty, and ambiguity. For the women, the fibromyalgia diagnosis validates and explains their symptoms and thus helps them understand their physical and emotional experiences; experiences, which for some participants have lasted for decades:

I could go back as early as my 20 years. I’m in my 40 years now but I would say as maybe as long ago as 20 years ago, maybe more, and it would explain, it would explain a lot, but I would say when did they, when did they get more pronounced and announced re-announced themselves severely? Probably in my mid-30s, roughly.

Angela

At this emotional level, the diagnosis represents a validation of experiences that does not require or warrant for further engagement at a scholarly level. Academics do not necessarily worry or take note of the cause of fibromyalgia. They just merely accept the fact they are sick and as such feel permitted to engage in life in whichever way is suitable and feasible for them. Unlike non-academic fibromyalgia patients who withdraw from employment in order to incorporate pacing as a coping mechanism (Brown, 2018), most academic participants ultimately decided to actively live with fibromyalgia, and to get on with life irrespective of and in spite of the fibromyalgia diagnosis.

I think you learn to live with the condition. I was being asked yesterday, “how has it affected you?” and I really have to think hard, how has it affected me? Because you forget, because you’ve learned to live with it, and have strategies to deal with what’s happening.

Dana

Through the objects, participants emphasise the permanence of fibromyalgia. They explain how the illness fluctuates and how symptoms move and change, but ultimately, their experience with fibromyalgia is shaped by its permanence, even in periods of remission. Despite the permanence that academics describe, the fluctuation in symptoms and severity coupled with the contested nature of fibromyalgia, also leave their marks on the participants. Some participants talk about doubting themselves, and being unsure regarding their diagnosis, whereas others talk about the body/mind split, in that the body would be more capable than the mind or vice versa:

I think at times it can feel like a battle between my body and mind, cause my mind can feel very active and good and my body does not at all, and it’s really unhappy, the more I try and do the less happy it becomes so it can feel like a battle.

Amy

It’s almost like having the imposter syndrome times two or squared, you know, that it’s; you have the regular imposter syndrome about being a writer and an academic and all that, but then I also have the imposter syndrome from do I really have an illness? Is it really real? Is it?

Yasmin

What was on the participants’ minds first and foremost, however, was the impact that living with fibromyalgia has on work and career. Although there are times where participants say they are more productive or sociable, their bad days and flare-ups mean that overall, they feel they are held back in what they could achieve without fibromyalgia, which leads to the process of redefining life goals.

### 3.2. The Process of Redefining Life Goals

Participants are acutely aware of the difference between what could and would have been, thus their virtual social identity [65] and what is, their actual social identity [65]. For academics this discrepancy between what is and what could be is a source for negative emotions, such as sadness and disappointment, but also anxiety and shame. In this sense, fibromyalgia makes academics discreditable [65].

It does mean being held back or slowed down […] I think if I hadn’t got fibro, oh yes, I would have gone up the academic chain quite quickly.

Bernie

I could have done much more, I could’ve done much more. […] I’ve had the school ask me, sometimes, ask me whether I would maybe cover a few sessions, maybe, and teach or cover a module for a semester […] I had to turn them down unfortunately and I had to say that I was flattered […] but I didn’t want to admit really, to them, that I wouldn’t have the energy to do it.

Kate

If somebody gave me a magic medicine right now that would stop the fibro. The next ten years of my career would look unbelievably different from the last ten years. If you looked at my CV. of what I’m able to achieve, just how slow I am versus other people […] if someone magically gave me a medicine that would take that block away and gave me 10 years out the gate to see what I could achieve, you know. Wind me up and watch me go because then is probably the only thing in the way right now, because of the stamina.

Sian

Although the women do not explicitly talk about Goffman’s theoretical treatises of stigma and spoiled identity [65], they do emphasise how they employ strategies to cope with this discrepancy between their virtual and actual social identities [65]. Whilst they acknowledge the impact of fibromyalgia, participants are concerned that the diagnosis of fibromyalgia does not become a self-fulfilling prophecy [66] or an excuse for being less productive or entirely unproductive at certain times, particularly as cognitive dysfunctions are seen as amongst the most intrusive of all fibromyalgia symptoms [67]. The academics in this study highlighted that fibromyalgia stopped them from achieving as much as they could, but were not willing to pace or give up on their academic work and academic life. At an emotional level, academics seek out the legitimacy to assume the sick role [68] and to withdraw from the academic rat-race. The academic engagement, the knowledge around the sick role, and indeed, the importance of academic work for their sense of self mean that they are not allowing themselves to indulge. Instead, they push themselves through difficult periods and bad days, potentially overcompensating for their perceived shortcomings. While non-academic women with fibromyalgia are able to situate themselves within their experiences of losses and gains [69], for academics, there is no silver lining or positive in the negative that is fibromyalgia [70].

### 3.3. The Refusal to Accept Fibromyalgia as a Diagnosis

Academics in this study do not embed their understanding of fibromyalgia into their lifestory and their beings; they incorporate society’s understanding into how they make sense of the condition at a cognitive level. How strongly academics have internalised society’s values and understanding of illness is evident in their personal interpretation of chronic conditions. Participants talked openly about being prejudiced and having biases against conditions like fibromyalgia. Their attitude towards fibromyalgia is such that they reject the contested fibromyalgia diagnosis:

I did fight against the diagnosis. I didn’t want to accept it, I didn’t want to believe that they’re actually…because it’s so like ME isn’t it? And those things. You don’t want to believe that, well I didn’t really want to believe I’ve got that because I didn’t think that was me.

Jackie

It’s Central Sensitisation Syndrome […] Central Sensitisation Syndrome is much more about my nervous system than fibromyalgia, and it was a malfunction in my nervous system where my nerves are forever all in a heightened state of alert.

Amy

I was diagnosed with Ehlers–Danlos syndrome. It’s a connective tissue disorder whereby collagen isn’t formed correctly […] if I’m filling out a form and it says, you know “do you have any medical conditions?” I will put down Ehlers–Danlos rather than fibromyalgia.

Hanna

Academics’ reluctance to accept a diagnosis that may feel uncomfortable is not unusual and represents a process of negotiation, what Madden and Sim [12] refer to as negotiated order within the diagnostic process. Academics are not so much trapped between their personal own bodies, culture, and society. They are fully entrapped by their academic minds, which prevents them from fully understanding and accepting fibromyalgia as a diagnosis. For them, a physical or psychological cause that has some demonstrable effect on the body and manifests itself as pain is much easier to grasp than the elusiveness of cognitive dysfunctions that seemingly appears out of nowhere. In truth, the academics’ engagement with fibromyalgia is not an entirely scholarly undertaking. It is a cerebral activity on their part to grasp and make sense of the condition, but their choice of readings and critiques highlights that the focus of their scientific, objective exploration remains firmly embedded in their personal experiences.

### 3.4. The Consideration of Identifying as a Patient

Academics had detailed knowledge of classifications of diseases. Some participants knew that the relevant International Classification of Diseases code clearly identifies fibromyalgia as a condition related to the musculoskeletal system and connective tissue within the group of the unspecified and otherwise unclassified disorders. For academics, therefore, the code describes fibromyalgia as a disease, but as a condition that is still categorised as unspecified and unclassified. Consequently, the process of being diagnosed and provided with that all-important classification of a specific illness is also met with ambivalence. Diagnosis is important as it provides a name and categorisation for the disabling symptoms individuals experience, whilst also reassuring them there is no terminal illness underlying the symptoms.

I still would rather know […] well, I don’t want anything sinister to be wrong, so it’s better to have that.

Jackie

Once I was diagnosed by a rheumatologist it all made sense and as far as feeling about it, it varies by day, it really does, it’s, I have mixed feelings about it, not always great, you know.

Angela

Academics also want reassurance, but are critical of doctors, who they see as gatekeepers to legitimise illness experiences [71]. For academics, the medical professionals are bound by medical discourses, which in the case of fibromyalgia are not easily discernible. Academics are aware of the roles that they and their medical professionals take within the doctor–patient relationship and understand the concepts of the sick role [68,72]. Within the scope of their reflections in relation to the process of diagnosis academics highlighted the responsibilities attached to a medical categorisation or diagnosis. In line with their understanding of the sick role, for the academics the fibromyalgia diagnosis comes with responsibilities, such as the responsibility of being a good patient. Being a good patient means being compliant and agreeable to treatments and of cooperating with the health care professionals.

Academics see engaging in treatments and cooperating with health care professionals as a necessary step to access the actual and actually relevant courses of treatment. Academics understand that it is the doctors’ responsibility to provide legitimacy [71], that the diagnosis enables the patient to be exempt from ordinary responsibilities, but that instead the patient is obliged to get better [68]. The participants even critiqued the roles we are all performing within the norms of society, and that by following these conventions and rules we are perpetuating this role division. The academics seem to miss that they themselves are bound within a biomedical view of their condition.

This is the first time I’ve been in a proper pain clinic, so that’s what,12 years it’s taken me to get into the room. […] It’s very affirmative seeing other people in the room of a variety of cultures, races, men, women, different sort of pain, and that’s really great, but that’s kind of it so far. So, I’m going and I’m doing, I’m being a good patient.

Alison

I’m never without the pain. So, but when it’s really bad at work it’s, I have my medication. I always make sure I have some in my drawer, some in my bag, I’m never without it. I would make sure that it’s there; and then the coffee as well. I have like cafetière with really strong coffee and I love the smell, just to keep me kind of, motivated.

Kate

At a rational level, they understand the social [73] and psychosocial factors [74] in relation to the meaning and experience of fibromyalgia. Yet, they seek out the medical professionals’ support to alleviate symptoms, thus to “fix the problem”. In this sense, their understanding of health is grounded in the expectation that they be free of symptoms. The academics feel that they are seen as difficult and problem patients, which they tie back to having fibromyalgia, a contested and contentious diagnosis. In their logic, they would not necessarily be able to get referred to the kinds of treatments they would potentially benefit from, if they were seen to be uncooperative. They think that by complying with the pain clinic schemes, for example, they are good patients and worthy of consideration for further developments and treatments.

## 4. Discussion

Fibromyalgia is associated with experiences of bias and stigmatisation [65], which are particularly severe before diagnosis because the variability and inconsistency of the symptomology cast doubt on the realness of the illness experience [75]. Knowing about the risk of being stigmatised results in the academics actively seeking to resist the marginalisation process on the basis of their condition. Academics resisting and employing resistance strategies represents nothing less than stages three—learning to cope—and four—learning to pass—in the moral career of the discredited or discreditable [65]. The way academics perform resistance differs from the resistance strategies of those diagnosed with chronic fatigue syndrome [37]. In people with chronic fatigue syndrome re-making the lifeworld manifests itself in the form of pacing strategies, the reduction of activities or the intensity with which activities are performed [37,76]. The re-making is embodied and bodily. The participants in this fibromyalgia study are re-making the lifeworld at a cerebral level through redefining their reality and creating a new, socially acceptable reality. This form of re-making resembles the normalisation processes [77,78] linked to maintaining identity continuity [79,80]. Academics perform their resistance at both, the scholarly, academic level and the personal, emotional level. They are overall reluctant to relinquish their academic work and continue with their ordinary activities pretending they are not ill. During the course of the study there were participants who were becoming too ill to continue in their current positions and roles. However, they were fully set upon continuing academic work and managing their illness and work by scaling back on hours and taking on slightly different positions. They were not willing to consider alternative careers or non-employment, which was an option for non-academics [69]. It may well be this attitude towards holding on to academic work that leads to their quasi-scholarly engagement with the condition. Engaging as an academic means to critique and question; and the participants do indeed critique and question the label, the diagnosis, the responses of others, but also their personal experience, their emotional responses. However, academics do not realise the pseudo-objective and quasi-scholarly nature of their endeavours to make sense of fibromyalgia and that they cannot truly separate the cerebral from the experiential. They do not see that they are holding on to medical models of illness and disabilities expecting the problem of fibromyalgia to be “fixed”. Yet, academics are also reluctant to use the fibromyalgia diagnosis to share their experiences with others within their social network, because they consider the diagnosis too difficult to explain and the condition too difficult to understand. This active rejection of the diagnosis represents another resistance strategy as a technique of information control [65] instead of admitting to and accepting the fibromyalgia diagnosis, which would be experienced as a threat to identity and sense of self.

### Limitations

No research is ever without its limitations, and, of course, research that spans epistemological, philosophical, and disciplinary conventions is often at risk of being seen as faulty because frameworks are misunderstood or misinterpreted. I am fully aware that my research could be seen as lacking robustness and validity because of the fluidity of participant recruitment or sampling. Additionally, my work may be criticised for its lack of details when it comes to measuring or quantifying pain experiences or identifying the exact description of symptomology of each individual participant. I understand that these are indeed limitations when viewed from a (bio)medical or psychological viewpoint. However, as this study was never aiming to be a (bio)medical or psychological treatise of the fibromyalgia experience, I would like to reiterate that these are not limitations to the sociological research undertaken.

Instead, I would like to focus on my choice of the theoretical lens as a limitation to this study. In many ways, the focus on the social course of illness by [37] enabled insights into individual experiences that other frameworks would not have been able to uncover. Yet, the specificity of the analytical framework in many ways limits our insights and produces a kind of tunnel vision of experiences amongst academics. More research is needed to definitively determine the relationship between personal distress and cultural expectations amongst academics with fibromyalgia and to then be able to compare that to members of the general public with fibromyalgia.

## 5. Conclusions

In this concluding section, I would like to return to the beginning, where I outlined the dual aim of this article: firstly, to highlight the difficulties of navigating an invisible and fluctuating chronic condition in an environment that emphasises productivity and excellence; and secondly, to shed light on strategies and techniques individuals employ to negotiate their lives with fibromyalgia and counteract marginalisation.

The social course of illness model by Ware [37] provided a fruitful point of departure for exploring and theorising the lived experience of academics with fibromyalgia. I have shown that academics with fibromyalgia try to make sense of their experiences and to learn living with the condition. At the same time, however, they struggle, and indeed, fail with both. As critical readers and thinkers, academics engage with definitions and treatments in ways that non-academics do not. As a result of this ability to critically reflect academics are fully aware of the contested nature of the condition and the stigma attached to it, which means they know of the marginalisation processes they are subjected to, and they therefore consciously decide to employ measures to counteract being marginalised. The four forms of resistance identified amongst academics with fibromyalgia are (1) the incorporation of cultural expectations and social conventions into personal life stories, (2) the re-making the lifeworld at a cerebral level through redefining reality and creating a new, socially acceptable reality, (3) active rejection of the fibromyalgia diagnosis, and (4) active and pro-active countermeasures to assuming the sick role.

It is noteworthy that there are similarities but significant differences between the resistance strategies Ware [37] identified and the experiences of academics with fibromyalgia presented in this study. It would be interesting now to explore the differences further. As highlighted in the introduction, according to Ware [37], the resistance strategies are a consequence of the marginalisation experienced based on individuals’ interpretation of their personal distress and the cultural expectations they feel subjected to. It appears that amongst academics both elements are aggravated, which may result in resistance processes that are significantly increased when compared to those individuals with fibromyalgia who are members of the general public. The original contribution to knowledge in this article lies in the identification of the four forms of resistance amongst the academics with fibromyalgia. The use of the sociological framework helps medical practitioners understand the complexity of lived experience and may also explain how and why individuals with fibromyalgia may not accept proposed treatments.

## Figures and Tables

**Table 1:** Summary of participants’ career status.

Pseudonyms	Career Stage	Employment	Institution
Alison	early career	temporary/hourly paid	HE
Amy	mid career	open-ended	HE
Angela	early career	open-ended	HE
April *	mid career	unemployed	not applicable
Bernie	mid career	open-ended	FE
Beth	mid career	temporary/hourly paid	FE
Calli	mid career	open-ended	HE
Carmen	early career	PhD GTA	HE
Dana	late career	open-ended	HE
Elena	early career	PhD GTA	HE
Erica	early career	PhD GTA	HE
Eryn *	early career	temporary/hourly paid	HE
Faith *	mid career	open-ended	HE
Hanna	early career	temporary/hourly paid	HE
Jackie	mid career	open-ended	HE
Jill	early career	temporary/hourly paid	HE
John	mid career	open-ended	HE
Joyce *	early career	unemployed	not applicable
Kate	mid career	open-ended	FE
Lana *	early career	unemployed	not applicable
Patricia *	late career	unemployed/retired	not applicable
Peg	mid career	open-ended	HE
Rebecca *	early career	temporary/hourly paid	HE
Scott	early career	freelance	not applicable
Sherry	late career	temporary/hourly paid	FE
Sian	early career	open-ended	HE
Tami *	early career	temporary/hourly paid	HE
Yasmin	mid career	open-ended	FE

* 8 participants, indicated by an asterisk, dropped out.

**Table 2:** Tabular introduction to levels and elements of interpretation within the Systematic Visuo-Textual Analysis [64].

	Element 1 Visual Only	Element 2 Textual Only	Element 3 Visuo-Textual Combined
Level 1 noticing and describing	Artistic in visual work (use of perspective, colour, space, form, tone, light, composition)	Linguistic in textual work (use of language, words, phrases, structure)	Connecting the visual and the textual (structure, meanings, expressions)
Level 2 conceptualising	Essential elements that unite artefacts	Words/phrases that capture patterns/themes	Connections between artefacts and themes

**Table 3:** Example of Systematic Visuo-Textual Analysis applied to Bernie’s data.

	Element 1 Visual Only	Element 2 Textual Only	Element 3 Visuo-Textual Combined
Level 1 noticing and describing	A backpack	Pain across the shoulders and across the hips heavy weight the tablets removed that weight, that pain you get from carrying a heavy backpack that backpack’s there. It is not going, that weight.	Location of pain type of pain experience negotiating and dealing with pain experience consistency and persistence of pain
Level 2 conceptualising	Physicality of weight and pressure on the body	Pain and fibromyalgia are always present Negotiating reality of pain being there	Making sense of experiences of pain Reality of accepting pain and condition, or not

**Table 4:** Example of Systematic Visuo-Textual Analysis applied to Sian’s data.

	Element 1 Visual Only	Element 2 Textual Only	Element 3 Visuo-Textual Combined
Level 1 noticing and describing	A soft, stretched out, old, grey, cotton T-shirt but that is crumbled up	Something you have had forever soft and colourless is kind of the way that having fibro feels like, not stabbing this dull thing trying to push it into a corner and forget about it, or crumple it up and throw it away	Consistency and persistence of pain type of pain experience type of pain experience negotiating and dealing with pain experience
Level 2 conceptualising	Physicality of weight and pressure	Pain and fibromyalgia are always present Negotiating reality of pain being there	Making sense of experiences of pain Reality of accepting pain and condition, or not

## Data Availability

Data cannot be publicly accessed and have not been archived for public use.
